# Critical Considerations for COVID-19 Vaccination of Refugees, Immigrants, and Migrants

**DOI:** 10.4269/ajtmh.20-1614

**Published:** 2021-01-13

**Authors:** Christine M. Thomas, Michael T. Osterholm, William M. Stauffer

**Affiliations:** 1 Division of Infectious Diseases and International Medicine, Department of Medicine, University of Minnesota, Minneapolis, Minnesota;; 2 Center for Infectious Diseases Research and Policy, University of Minnesota, Minneapolis, Minnesota;; 3 Center for Global Health and Social Responsibility, University of Minnesota, Minneapolis, Minnesota

## Abstract

As COVID-19 vaccines are distributed across the United States, it is essential to address the pandemic’s disproportionate impact on refugee, immigrant, and migrant (RIM) communities. Although the National Academies Press *Framework for Equitable Allocation of COVID-19 Vaccine* provides recommendations for an equitable vaccine campaign, implementation remains. Practical considerations for vaccine rollout include identifying and overcoming barriers to vaccination among RIM communities. To identify barriers, information regarding vaccine beliefs and practices must be incorporated into the pandemic response. To overcome barriers, effective communication, convenience of care, and community engagement are essential. Taking these actions now can improve health among RIM communities.

COVID-19 vaccine distribution is imminent, and U.S. refugee, immigrant, and migrant (RIM) populations, who are disproportionately affected by COVID-19, face well-known barriers to vaccination. If not addressed, these barriers likely will result in a lost opportunity to save lives. The recent report from the National Academies Press, *Framework for Equitable Allocation of COVID-19 Vaccine* (*Framework*), offers specific and helpful recommendations for conducting an equitable vaccine campaign,^1^ although implementation remains. Federal, state, and local public health agencies must prioritize the *Framework* recommendations, consider the practical implications for implementation, and plan to overcome anticipated barriers.

Refugee, immigrant, and migrant populations include more than 40 million people living in the United States who were born in another country.^2^ This diverse group represents hundreds of cultures and languages, with some groups and individuals newly arrived, and others well established. Motivations for migration range widely, with some fleeing persecution, whereas others are joining family, attaining higher education, or pursuing economic opportunity in various sectors (e.g., seasonal farm labor, technology, and health care). Within the United States, these communities represent a broad range of socioeconomic status, educational attainment, occupations, and English proficiency, and are integral to the nation’s social, cultural, and economic vitality. Although each community is unique, many are heavily represented in essential industries which, added to barriers surrounding public health and medical services, contribute to a disproportionate impact from the COVID-19 pandemic.

Compounding the pandemic’s impact, many RIM communities experience barriers to vaccination even under normal circumstances, resulting in lower vaccination rates than in people born in the United States.^3^ While the *Framework* advocates for inclusion of these groups, hurdles to implementation are immense. As the *Framework* is an exhaustive 250-page document, with the most important information regarding RIM communities in the latter half, key information may not be noticed. To reach the intended audience and have timely impact, this key information must be prioritized and effectively distributed, acknowledged, and utilized at the state and local community levels. To increase adoption of the *Framework* recommendations, state and local health departments must dedicate personnel time and resources to ensure that recommendations are integrated into planning processes.

Given limited information regarding knowledge, attitudes, and practices (KAP) surrounding vaccines, historic mistrust in certain communities, a turbulent U.S. political environment, and novelty of COVID-19 and associated vaccines, heightened vaccine hesitancy can be anticipated. To overcome these and associated barriers, information and data are desperately needed in real time for RIM communities. Immediate collection and sharing of KAP and other data must be undertaken, shared, and incorporated into the pandemic response to inform vaccine messaging, planning, and distribution. Consider, for example, the lack of data on COVID-19 vaccine beliefs and practices in RIM communities. A survey representative of the U.S. population reported that less than 60% of adults endorsed an intent to receive a COVID-19 vaccine.^4^ Although in this study younger age, black race, lower formal educational obtainment, and not receiving last year’s influenza vaccine were associated with lack of vaccination intent,^4^ there are no published data indicating if these or other factors might be associated with COVID-19 vaccine hesitancy in RIM communities.

Refugee, immigrant, and migrant populations are not homogeneous, and communities vary greatly in their experience with, and attitudes toward, vaccination. There may be useful lessons from vaccination efforts during the H1N1 pandemic, when a seemingly paradoxical trust of vaccines in general, but strong hesitancy toward the H1N1 vaccine stemming from concerns over vaccine novelty and safety, was found among hard-to-reach Latinx communities.^5^ There may also be specific vaccine factors (e.g., purported inclusion of pork products) or misinformation (e.g., purported risk of autism) that make a vaccine more or less acceptable to certain groups. Overall, barriers to vaccination faced by RIM communities include cultural factors, differing understanding and beliefs of disease processes, healthcare utilization challenges (e.g., accessing care and system familiarity), limited trust (particularly of government entities), and specific vaccine concerns (efficacy, necessity, and safety).^5,6^

Practical approaches to reduce vaccination barriers among RIM communities must be incorporated before and during state and local vaccine rollout, and include effective communication and convenience of care. The *Framework* highlights that individuals should receive vaccine information in a primary language, in a culturally appropriate manner, and from a trusted source. These recommendations present challenges, since methods and effectiveness of communication differ by format (e.g., oral versus written), distribution (e.g., radio, television, and social network platforms), and perceived source trustworthiness (e.g., friends, religious or community leaders, and employers). Also essential is ensuring vaccination convenience by bringing vaccines to people, rather than people to vaccines. This can reduce barriers surrounding healthcare access (e.g., competing priorities such as time away from work, fear or mistrust of authorities, and transportation challenges).

Perhaps, the *Framework*’s most important recommendations are that communities be engaged directly and that vaccination efforts use community partners who have trusted relationships. Unfortunately, there has been a historic lack of investment by many governmental bodies and public health agencies in developing and maintaining true community partnerships. Integrating and welcoming community groups and members into the process is vital and can provide invaluable allies in building trust and relationships, guiding communication and logistics, and providing critical feedback. Ideally, these working relationships would be ongoing and exist before a public health emergency. However, if these relationships do not exist, it is imperative that public health agencies immediately seek out partnerships with community-based organizations that possess trusted relationships with their respective communities. True partnerships also involve community representation in the workforce and at the leadership and decision-making level. This may be accomplished by training and engaging community workers and by creating a formal relationship such as a community advisory board (CAB), which may help guide intervention development and implementation. A CAB can be invaluable in decision-making processes, through advising about community priorities and logistics, connecting with trusted leaders and groups, providing insight about behaviors or challenges, and relaying feedback from the community as the work unfolds.

To aid in these efforts, a National Resource Center for COVID-19 Contact Tracing, Mitigation, and Prevention in RIM (NRC-RIM) is being established with the intent to support state and local health departments through the collection and dissemination of data, centralization of best and promising practices/models for reaching and working with RIM communities, and development of linguistically and culturally appropriate communication materials. The NRC-RIM will also offer direct support and guidance such as assistance with establishing a CAB and models and tool kits for partnership building between health departments and community organizations: https://nrcrim.umn.edu.^7^

Development of the *Framework* to address anticipated inequities in COVID-19 vaccination programs in the United States is a welcome and essential first step, but the hardest work of implementation by public health agencies remains. It is essential that *Framework* recommendations be acknowledged, prioritized, and incorporated. Crucial next steps in decreasing disparities, increasing vaccination success, and ending the pandemic must be taken immediately, and include 1) distribution of the *Framework* among public health agencies, dedicating resources for implementation; 2) collection and dissemination of data (e.g., KAPs, sharing successful models, and promising practices); 3) implementation and sharing of practical solutions to barriers faced by RIM populations (e.g., communication materials and vaccine accessibility models); and 4) community engagement (e.g., outreach to community-based organizations with trusted relationships, training, and hiring of persons from the community to serve the community; inclusion of community voices in leadership and planning; and execution of vaccination efforts through liaisons or CABs).

Globally, many RIM populations are at increased risk of COVID-19 infection and death due to factors such as ongoing stigma and discrimination, economic disenfranchisement, and barriers to public health and medical care services. Three-quarters of the world’s refugees and migrants are hosted in regions of the world that are considered under-resourced,^8^ and equitable care delivery, including vaccines, should be paramount. Throughout the COVID-19 pandemic, agencies including the WHO, United Nations, and International Organization for Migration have sought to bring attention and resources to the inequities and impending crises faced by many RIM communities.^8,9^ Although the *Framework* was developed for U.S. vaccine allocation, many recommendations may be relevant for equitable vaccine distribution among RIM populations throughout the world. In addition, the practical considerations for implementation mentioned here are not specific to the United States and, if applied globally, may assist in a “coherent, effective international approach that leaves no-one behind.”^8^

It is essential that RIM communities are acknowledged as a distinct subgroup of those disproportionately affected by COVID-19. The *Framework* provides excellent considerations for ensuring equitable access to COVID-19 vaccination among RIM communities. Public health agencies tasked with implementing COVID-19 vaccine rollout among these communities should use the *Framework*’s guidance, seek to understand the community’s vaccine beliefs and practices, and reduce barriers to vaccination by intentionally partnering with the community. Developing and sharing resources, practical strategies, and best and promising practices will improve the health of RIM communities during the pandemic and into the future. These actions need immediate attention. If implemented well, effective vaccination will prevent many COVID-19 illnesses and deaths in RIM communities and offer progress toward a more equitable social and health paradigm that includes RIM populations in policy and implementation planning.
